# Gfa1-dependent hexosamine metabolism maintains cell-wall integrity and developmental fitness required for crop colonization in *Aspergillus flavus*

**DOI:** 10.1016/j.tcsw.2026.100184

**Published:** 2026-10-15

**Authors:** Qijian Qin, Bin Wang, Wenxia Fang

**Affiliations:** a Institute of Biological Sciences and Technology, Guangxi Academy of Sciences, Nanning, 530007, China; b State Key Laboratory of Non-food Biomass Energy Technology, Nanning, Guangxi, 530007, China

**Keywords:** *Aspergillus flavus*, Gfa1, Hexosamine biosynthetic pathway, Cell wall, Chitin, Sclerotium, Crop colonization

## Abstract

The fungal cell wall is a dynamic structure whose composition and integrity are closely coupled to metabolism, morphogenesis, and environmental fitness. Glutamine fructose-6-phosphate amidotransferase (Gfa1), the committed enzyme of the hexosamine biosynthetic pathway, supplies precursors for UDP-*N*-acetylglucosamine production and cell-wall chitin synthesis. Although Gfa1 is essential for growth and virulence in *Aspergillus fumigatus*, its role in the cell-wall biology and developmental program of the aflatoxigenic fungus *Aspergillus flavus* remains unclear. Here, we characterized a *gfa1* deletion mutant and a complemented strain of *A. flavus*. Loss of *gfa1* caused glucosamine/*N*-acetylglucosamine auxotrophy and markedly impaired vegetative growth and conidiation. Despite amino-sugar supplementation, the mutant exhibited increased sensitivity to cell-wall perturbing agents and significant reductions in cell-wall chitin and glucan, indicating disrupted cell-wall homeostasis. Gfa1 deficiency also severely impaired sclerotial formation and increased sensitivity to several environmental stresses. Importantly, the mutant showed markedly reduced colonization of peanut and maize seeds and no detectable aflatoxin B1 accumulation under the conditions tested. Consistent with its broad fitness defects, Δ*gfa1* displayed strongly attenuated virulence in *Galleria mellonella*. These findings establish Gfa1-dependent hexosamine metabolism as an important determinant of cell-wall homeostasis and developmental competence in *A. flavus* and reveal a link between amino-sugar metabolism, fungal surface integrity, sclerotial differentiation, and crop-associated fitness.

## Highlights

• Gfa1 is required for amino-sugar-dependent growth in *Aspergillus flavus*.
• Gfa1 deficiency disrupts chitin, glucan, and cell-wall integrity.
• Gfa1-dependent metabolism is required for sclerotial development.
• Loss of Gfa1 impairs colonization of peanut and maize seeds.
• Gfa1 deficiency prevents detectable AFB1 accumulation during colonization.

## 1 Introduction

The fungal cell wall is a dynamic extracellular structure that provides mechanical support while coordinating morphogenesis, environmental adaptation, and interactions with host tissues (Gow, 2025; Shree et al., 2024). In filamentous fungi, the cell wall is composed predominantly of polysaccharides, including β-glucans and chitin, together with glycoproteins and other associated components (Garcia-Rubio et al., 2020; Ost et al., 2025). Rather than functioning as an inert exoskeleton, the fungal cell wall undergoes continuous remodeling in response to developmental and environmental cues and is therefore closely integrated with cellular metabolism and signaling (Latgé, 2007; Gow et al., 2017; Cortés et al., 2019).

*Aspergillus flavus* is an ecologically versatile filamentous fungus with major implications for both human health and food security (Hedayati et al., 2007; Abdullaziz et al., 2026). It is an important opportunistic pathogen and a major producer of aflatoxin B1 (AFB1), a potent carcinogen that can contaminate maize, peanut, and other crops (Klich, 2007; Amaike and Keller, 2011; Cary et al., 2018). *A. flavus* also undergoes a distinctive developmental program that includes conidiophore formation and sclerotial differentiation, specialized developmental processes that contribute to dissemination and environmental persistence (Horn et al., 2009; Cho et al., 2022). Successful completion of these processes requires coordinated regulation of metabolism, cell-wall assembly, and morphogenesis.

Chitin is an important structural component of the *Aspergillus* cell wall and is synthesized using UDP-*N*-acetylglucosamine (UDP-GlcNAc) as its activated sugar donor (Gastebois et al., 2009; Gow et al., 2017). UDP-GlcNAc also contributes to protein glycosylation and other cellular processes, placing amino-sugar metabolism at an important interface between primary metabolism and cell-surface organization (Lenardon et al., 2010; Paneque et al., 2023). The hexosamine biosynthetic pathway (HBP) provides the principal endogenous route for UDP-GlcNAc production in fungi (Milewski et al., 2006; Paneque et al., 2023). Gfa1, also known as glutamine fructose-6-phosphate amidotransferase, catalyzes the committed step of this pathway by converting fructose-6-phosphate and glutamine to glucosamine-6-phosphate (Milewski, 2002).

The importance of Gfa1-dependent amino-sugar metabolism has been demonstrated in several fungi. In *Saccharomyces cerevisiae*, GFA1 is essential and its increased expression can enhance chitin synthesis (Lagorce et al., 2002). In *Aspergillus niger*, disruption of *gfaA* causes glucosamine or *N*-acetylglucosamine auxotrophy and impairs spore germination (Ram et al., 2004). Downstream HBP enzymes, including Gna1, Agm1 and Uap1, are also required for growth and virulence in *A. fumigatus* (Fang et al., 2013a, Fang et al., 2013b; Lockhart et al., 2020).

Recent work has established Gfa1 as an important determinant of *A. fumigatus* fitness. Loss of *gfa1* causes amino-sugar auxotrophy and, under partial repression conditions, results in defects in growth, conidiation, germination, cell-wall integrity, and stress adaptation, together with attenuated virulence in infection models (Qin et al., 2025). These findings are consistent with the phenotypes observed in the present study of *A. flavus*, supporting a conserved role for Gfa1-dependent hexosamine metabolism in fungal growth and fitness across *Aspergillus*. However, the biological functions of Gfa1 in *A. flavus* have not been systematically characterized.

*A. flavus* has a distinct ecological and developmental lifestyle characterized by sclerotial differentiation, colonization of oil-rich crops, and aflatoxin production (Amare and Keller, 2014; Ohkura et al., 2018). These species-specific traits raise the possibility that Gfa1-dependent amino-sugar metabolism may contribute not only to fungal growth but also to developmental differentiation and adaptation to crop-associated environments. In particular, the relationship between hexosamine metabolism, cell-wall homeostasis, and developmental fitness in *A. flavus* remains poorly understood. Because UDP-GlcNAc supplies the substrate for chitin synthesis and contributes to protein glycosylation and cell-surface-associated processes, disruption of the HBP could affect both cell-wall organization and developmental competence.

Here, we investigated the contribution of Gfa1-dependent hexosamine metabolism to the cellular and developmental fitness in *A. flavus*. Using targeted gene deletion and genetic complementation, we characterized the effects of Gfa1 deficiency on amino-sugar dependence, fungal growth and development, cell-wall integrity and composition, environmental stress responses, sclerotial formation, crop-seed colonization, AFB1 accumulation, and virulence. Our results demonstrate that Gfa1 is required not only for fungal growth but also for maintaining cell-wall homeostasis and developmental competence, thereby linking amino-sugar metabolism to the environmental and crop-associated fitness of *A. flavus*.

## 2 Materials and methods

### 2.1 Fungal strains and culture conditions

The strains used in this study included the *A. flavus* parental strain (CA14Δ*ku70*Δ*pyrG* and CA14Δ*ku70*), the *gfa1* deletion mutant (Δ*gfa1*), and the complemented strain (RT). Standard cultivation was performed at 28 °C or 37 °C on minimal medium (MM). The MM contained 1% (wt/vol) glucose, 0.092% (wt/vol) ammonium tartrate dibasic, 20 μL/mL salt solution, and 1 μL/mL trace element (TE). Solid plates were prepared by adding 1.2% agar. One liter salt solution was composed of 26 g KCl, 76 g KH_2_PO_4_ and 26 g MgSO_4_·7 H_2_O. The 100 mL TE solution was composed of 0.04 g Na_2_B_4_O_7_·10 H_2_O, 0.4 g CuSO_4_·5 H_2_O, 0.8 g MnSO_4_·4 H_2_O, 0.8 g Na_2_MoO_4_·10 H_2_O, 8 g ZnSO_4_·7 H_2_O and 0.81 g FeCl_3_. Where required, MM was supplemented with uracil (1.1 μg/ml) and uridine (1.2 μg/ml) to support the auxotrophic parental strain. Because the Δ*gfa1* mutant requires exogenous amino sugar for growth, glucosamine (GlcN) was supplied where indicated. Short-term stocks were maintained at 4 °C, and long-term stocks were stored at −80 °C in 25% glycerol.

### 2.2 Identification and domain analysis of Gfa1

The *A. flavus* NRRL3357 genome was searched using the *S. cerevisiae* Gfa1 protein (GenBank accession NP_012818) as the query. Conserved domains in the predicted *A. flavus* Gfa1 protein were identified using SMART.

### 2.3 Construction and verification of Δ*gfa1* and complemented strains

The *gfa1* deletion mutant was generated by homologous recombination using approximately 1.0-kb upstream and downstream regions flanking the target locus. The selectable *pyrG* marker was fused with the flanking regions by fusion PCR. The resulting deletion cassette was introduced into protoplasts of the auxotrophic parental strain. Transformants were selected on regeneration medium supplemented with GlcN and verified by diagnostic PCR. For complementation, a construct containing the native *gfa1 l*ocus and appropriate flanking sequences was generated by fusion PCR and introduced into Δ*gfa1* protoplasts. Transformants were selected under conditions permitting growth only after restoration of prototrophy. Correct integration was confirmed by PCR using independent primer pairs.

### 2.4 Amino-sugar rescue and carbon-source utilization

Freshly harvested conidia were serially diluted and spotted onto MM containing 1% (wt/vol) individual carbon sources, including GlcN, GlcNAc, glucose, fructose, mannose, maltose, lactose, galactose, arabinose, xylose, glycerol, and ethanol. To examine amino-sugar-mediated rescue, conidia were spotted onto MM containing different concentrations of glucose (0.55, 2.75, 5.5, 27.5, and 55 mM) in combination with 0, 1, 5, 10, or 50 mM GlcN. Plates were incubated at 37 °C for 48 h and photographed.

### 2.5 Growth, conidiation, conidiophore, and sclerotial development

For growth analysis, 10^6^ conidia were point-inoculated onto MM containing 2.75 mM glucose and 5 mM GlcN and incubated at 28 °C or 37 °C. Colony diameters were measured along two perpendicular axes at 24 h intervals. Conidia were harvested by flooding plates with 0.2% Tween 20 and quantified using a hemocytometer. Conidiophore development was examined microscopically after lactophenol cotton blue staining. For sclerotial formation, 10^6^ conidia were inoculated onto the same medium and incubated in darkness at 37 °C. Colonies were photographed before and after washing with 75% ethanol to remove aerial hyphae and conidia and expose sclerotia.

### 2.6 Cell-wall stress sensitivity and cell-wall composition

Conidial suspensions were spotted onto MM containing 2.75 mM glucose and 5 mM GlcN supplemented with the indicated concentrations of Calcofluor White (CFW), Congo red (CR), caspofungin, sorbitol, TCEP (tris(2-carboxyethyl)phosphine), or FeSO_4_. Plates were incubated under the indicated conditions before imaging. For cell-wall composition analysis, mycelia were harvested after growth in liquid MM containing 2.75 mM glucose and 5 mM GlcN.

Cell-wall material was prepared according to François (2006), followed by acid hydrolysis and monosaccharide analysis by HPAEC-PAD using a CarboPac PA-10 analytical column equipped with an AminoTrap guard column. The separation was performed isocratically with 18 mM NaOH at a flow rate of 1 mL/min. Monosaccharide standards, including glucose, glucosamine (GlcN), mannose, and galactose, were prepared at multiple concentrations and analyzed under the same chromatographic conditions as the cell-wall hydrolysates. Glucose was used as the reference standard for the total glucan fraction (α-and β-glucans), GlcN for chitin, mannose for mannan, and galactose for galactomannan (GM). Standard curves generated from these reference monosaccharides were used for quantitative determination of the corresponding cell-wall polysaccharide components.

### 2.7 Temperature and environmental stress assays

Growth was compared at 28 °C, 37 °C, and 42 °C. Relative growth inhibition at 28 °C and 42 °C was calculated relative to growth at 37 °C. Stress sensitivity at 37 °C or 28 °C was assessed using the indicated agents. Sorbitol was used as an osmotic stressor to assess the ability of the mutant to maintain cellular homeostasis under altered osmotic conditions. TCEP was included as a reducing agent to perturb the cellular redox environment and redox-sensitive protein processes, thereby testing the capacity of the mutant to maintain redox homeostasis. FeSO₄ was used to challenge iron homeostasis by increasing the availability of ferrous iron and to assess the cellular response to altered iron conditions. Because ferrous iron can also participate in redox reactions, the effects of FeSO₄ may reflect both altered iron homeostasis and associated redox perturbation.

### 2.8 RNA isolation and quantitative real-time PCR

*A. flavus* was grown for 24 h and treated with 100 μg/ml CFW or CR for 0, 30, and 60 min. Total RNA was extracted using the TransZol Up Plus RNA Kit (TransGen, Beijing, China) and reverse-transcribed using the HiScript II Q RT SuperMix for qPCR (Vazyme, Nanjing, China). qPCR was performed with ChamQ Universal SYBR qPCR Master Mix (Vazyme, Nanjing, China) on an ABI 7500 System using gene-specific primers (Table S1), with cycling conditions of 95 °C for 10 s and 60 °C for 30 s for 40 cycles. Relative expression was calculated using the 2^−∆∆CT^ method (Livak and Schmittgen, 2001) with β-actin as the internal control. Samples were tested in triplicate.

### 2.9 Peanut and maize seed colonization

Peanut and maize seeds of uniform size were surface-disinfected sequentially with 0.05% sodium hypochlorite for 3 min and 75% ethanol for 1 min. Seeds were rinsed three times with sterile water and inoculated with 10^6^ conidia of WT, Δ*gfa1*, or RT strains. Infected seeds were incubated at 28 °C in darkness for 6 days under humid conditions. After incubation, seeds were transferred to tubes containing 20 mL of 0.2% Tween 20 and vortexed for 5 min to dislodge conidia. Serial dilutions were prepared and conidia were quantified using a hemocytometer.

### 2.10 Aflatoxin B1 detection

AFB1 was extracted from infected peanut and maize samples using the procedure (Usman et al., 2022). Extracts were partitioned with chloroform, evaporated to dryness, and redissolved in chloroform. Samples were separated by TLC using chloroform (9:1) and visualized under UV illumination at 365 nm.

### 2.11 *Galleria mellonella* infection assay

Sixth-instar *G. mellonella* larvae were randomly divided into four groups of 90 larvae. Each larva was injected into the hind proleg with 10 μL of a suspension containing 10^4^ conidia of the WT, Δ*gfa1*, or RT strain using a Hamilton syringe. Control larvae received 0.2% Tween 20. Larvae were incubated at 37 °C and mortality was recorded at 24 h intervals.

### 2.12 Statistical analysis

Data were analyzed using GraphPad Prism 8 and are presented as mean ± SD. Statistical tests were selected according to experimental design. One-way ANOVA followed by an appropriate multiple-comparison test was used for comparisons involving more than two groups, whereas appropriate pairwise tests were used for two-group comparisons. The exact statistical test and number of independent biological replicates are indicated in the corresponding figure legends.

## 3 Results

### 3.1 Gfa1 is required for endogenous amino-sugar supply and normal growth of *A. flavus*

A BLASTp search of the *A. flavus* NRRL3357 genome using *S. cerevisiae* Gfa1 identified a single putative ortholog, encoded by G4B84_002808 (QMW27519.1). The *A. flavus gfa1* locus spans 2408 bp and contains six exons and five introns, with a 2082-bp coding sequence encoding a 693-amino-acid protein. SMART analysis identified two sugar isomerase domains and one glutamine amidotransferase domain, consistent with the conserved architecture of fungal Gfa1 proteins (Fig. 1A).

**Fig. 1:**
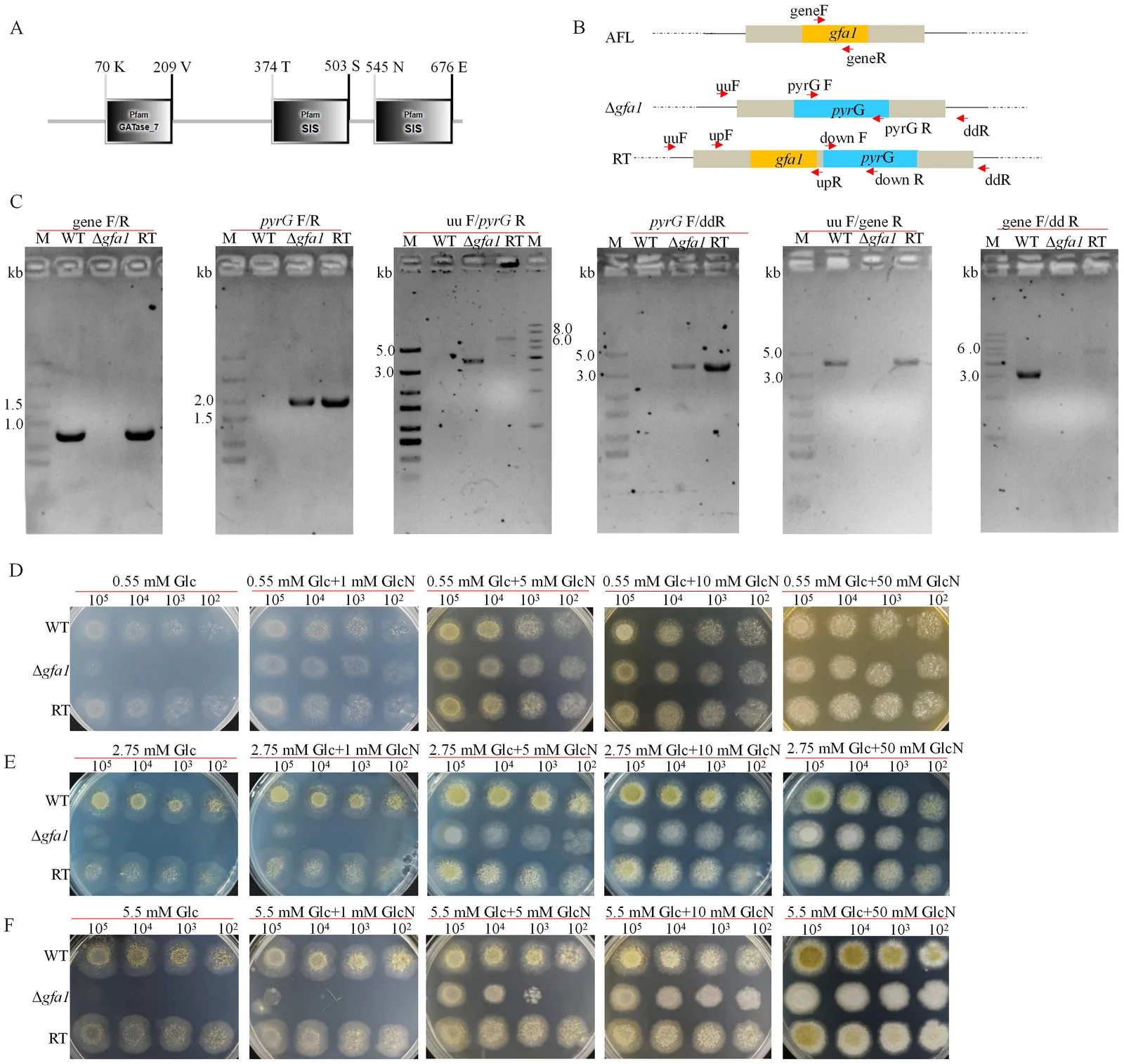
Domain architecture of Gfa1, construction of the Δ*gfa1* and RT strains, and growth of the Δ*gfa1* mutant on minimal medium containing different concentrations of Glc and GlcN. (A) Domain prediction of the Gfa1 protein using SMART. SIS, sugar isomerase domain; GATase, glutamine amidotransferase domain. (B) Schematic representation of the strategy used to generate the Δ*gfa1* mutant and the RT strain *via* homologous recombination. (C) PCR confirmation of the Δ*gfa1* mutant and RT strains using the six primer pairs indicated in (B). WT, wild-type; Δ*gfa1*, deletion mutant; RT, revertant strain. (D–F) Serial dilutions of conidia (10^2^ to 10^5^) from the WT, Δ*gfa1*, and RT strains were spotted onto MM supplemented with the following combinations of carbon sources: (D) 0.55 mM Glc with 1 to 50 mM GlcN, (E) 2.75 mM Glc with 1 to 50 mM GlcN, and (F) 5.5 mM Glc with 1 to 50 mM GlcN. Plates were incubated at 37 °C and photographed after 48 h. Three biological replicates were performed.

We generated a *gfa1* deletion mutant by homologous recombination and restored the wild-type *gfa1* locus in a complemented strain (RT) (Fig. 1B). Diagnostic PCR confirmed the expected genetic configurations of the Δ*gfa1* and RT strains (Fig. 1C). All primers used in this study are listed in Table S1. The Δ*gfa1* mutant failed to grow on MM containing glucose, fructose, mannose, glycerol, ethanol, or other tested carbon sources, whereas robust growth was observed when GlcN or GlcNAc was supplied as the carbon source (Fig. S1). Thus, loss of *gfa1* rendered *A. flavus* dependent on exogenous amino sugars.

Because Gfa1 catalyzes the first committed step of the hexosamine biosynthetic pathway, we reasoned that exogenous GlcN could bypass the metabolic block caused by *gfa1* deletion by supplying a downstream amino-sugar precursor. We therefore examined whether GlcN could restore growth of the Δ*gfa1* mutant when glucose was provided as the principal carbon source. Testing a range of glucose and GlcN concentrations allowed us to assess the extent of metabolic rescue and to establish appropriate conditions for subsequent phenotypic analyses. Addition of GlcN restored Δ*gfa1* growth at low glucose concentrations. The strongest recovery was observed with 0.55 mM glucose plus 10 mM GlcN, 2.75 mM glucose plus 5 mM GlcN, or 5.5 mM glucose plus 1 mM GlcN (Fig. 1D–F). In contrast, glucose concentrations above 27.5 mM prevented detectable growth rescue even when GlcN was supplied at 50 mM (Fig. S2). We therefore used 2.75 mM glucose plus 5 mM GlcN for subsequent phenotypic analyses. Under these amino-sugar-supported conditions, Δ*gfa1* remained substantially impaired in vegetative growth compared with WT and RT strains (Fig. 2A and B), indicating that exogenous GlcN provides a metabolic bypass of the Gfa1-dependent step and partially restores the growth of the Δ*gfa1* mutant under the conditions tested; however, the mutant remains substantially impaired compared with the WT strain.

**Fig. 2:**
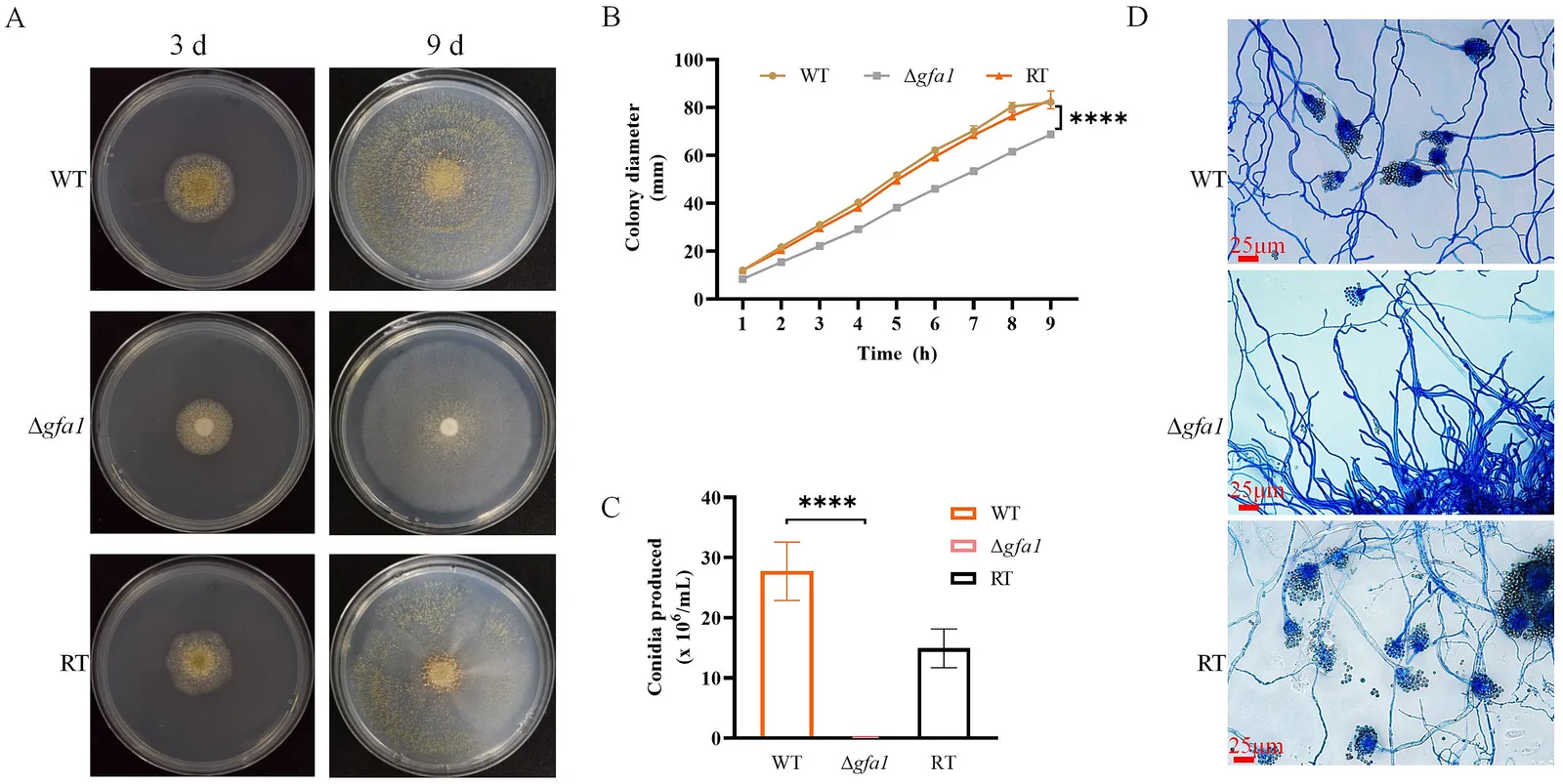
Growth and conidiation of the WT, ∆*gfa1*, and RT strains on minimal medium supplemented with 2.75 mM glucose and 5 mM GlcN. (A) Conidia of the WT, ∆*gfa1*, and RT strains were point-inoculated onto minimal medium supplemented with 2.75 mM glucose and 5 mM GlcN and incubated at 37 °C for 3 and 9 days. (B) Colony diameters of the three strains grown on the same medium were measured daily over a 9-day period. Data are presented as mean ± SD from three biological replicates. (C) Conidial production was quantified using a hemocytometer after 9 days of incubation on the same medium at 37 °C. Values represent the mean ± SD; statistical significance was determined by One-way ANOVA multiple-comparison test (****, *P* < 0.0001). Three biological replicates were performed. (D) Conidiophore morphology of the WT, ∆*gfa1*, and RT strains was examined microscopically after staining. Scale bar is 25 μm.

### 3.2 Gfa1 deficiency strongly impairs conidiation and conidiophore development

To further investigate the functional role of *gfa1*, conidia of the wild-type (WT), Δ*gfa1*, and revertant (RT) strains were spot-inoculated onto minimal medium supplemented with 2.75 mM Glc and 5 mM GlcN to support mutant growth. As shown in Fig. 2, the Δ*gfa1* mutant displayed a pronounced defect in asexual development. After 9 days of incubation, conidial production was reduced approximately 199.5-fold relative to WT and 107.5-fold relative to RT (Fig. 2C). Microscopic examination further revealed substantially fewer conidiophores in the mutant than in WT and RT strains (Fig. 2D). These findings indicate that Gfa1-dependent amino-sugar metabolism is required for efficient conidiophore formation and conidiation in *A. flavus*.

### 3.3 Gfa1 maintains cell-wall integrity and polysaccharide homeostasis

Because Gfa1 catalyzes the rate-limiting step of the hexosamine biosynthetic pathway and generates precursors required for chitin biosynthesis, we first examined whether Gfa1 deficiency altered cell-wall integrity. Compared with WT and RT strains, Δ*gfa1* showed markedly increased sensitivity to the cell-wall perturbing agents CFW, CR, and caspofungin at 37 °C (Fig. 3A). Notably, supplementation with exogenous GlcN at a concentration sufficient to support growth of the Δ*gfa1* mutant did not substantially alleviate these cell-wall defects. Thus, restoring extracellular amino-sugar availability was insufficient to fully restore cell-wall integrity in the absence of Gfa1.

**Fig. 3:**
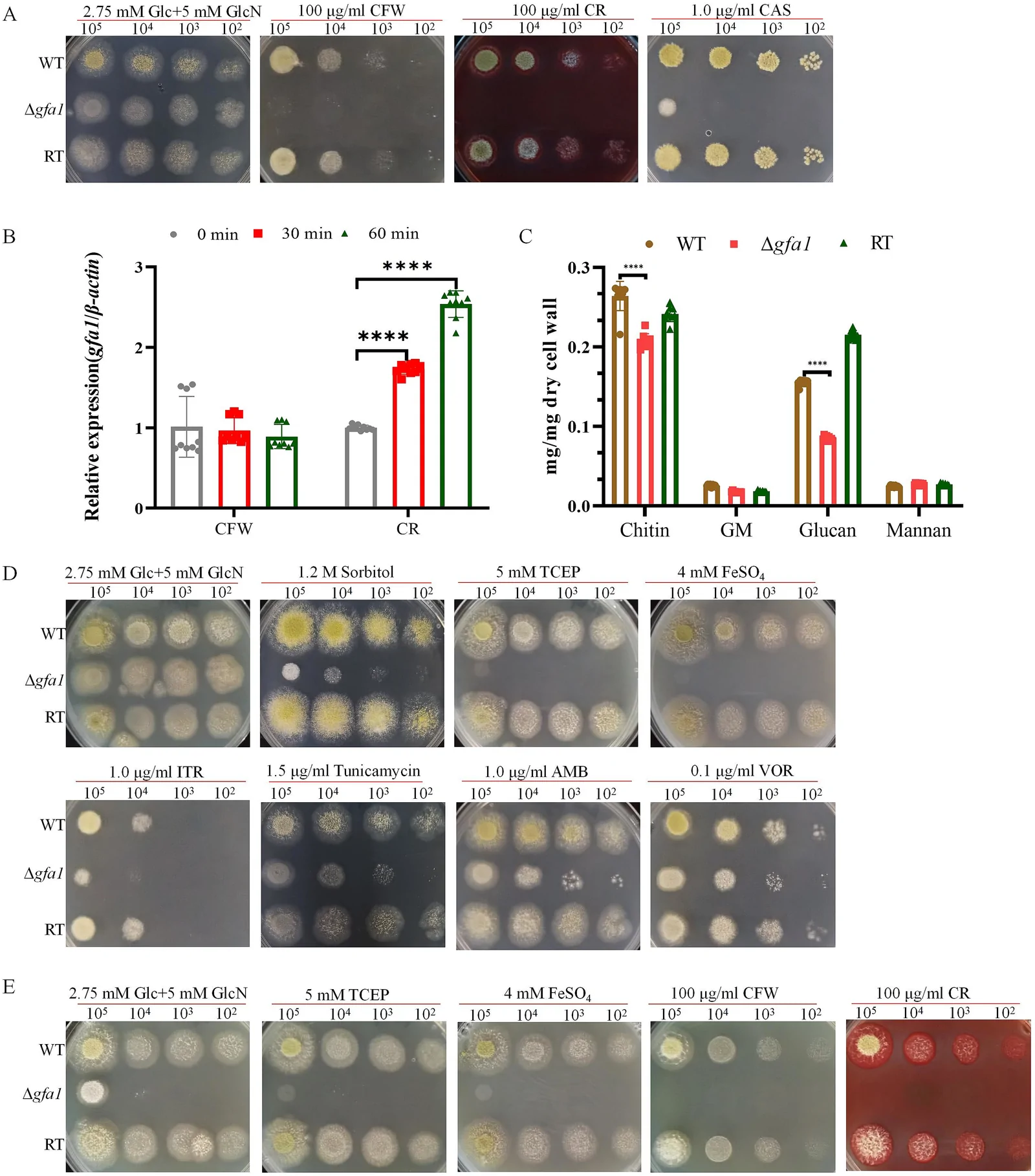
Responses of the Δ*gfa1* mutant to various stresses. (A, D, E) Serial dilutions of conidia (10^2^–10^5^) from the WT, Δ*gfa1* mutant, and RT strain were spotted onto MM containing 2.75 mM Glc and 5 mM GlcN plus the indicated stress agents, and incubated at 37 °C for 48 h (A, D) or at 28 °C for 48 h (E). (B) Transcriptional response of *gfa1* to cell-wall stress. WT mycelia grown for 24 h were treated with 100 μg/ml CFW or Congo red for 0, 30, and 60 min, and relative *gfa1* expression was determined by RT-qPCR. Data are mean ± SD from three independent experiments. (C) Cell wall components were quantified from 10 mg dry mycelia after growth in MM containing 2.75 mM Glc and 5 mM GlcN at 37 °C for 48 h. Three biological replicates were performed. Data are mean ± SD; one-way ANOVA with multiple-comparison test (****P < 0.0001). Glucan, total glucan including α- and β-glucans; GM, galactomannan. (For interpretation of the references to colour in this figure legend, the reader is referred to the web version of this article.)

We next asked whether *gfa1* expression is regulated in response to cell-wall stress. WT mycelia grown for 24 h were treated with CFW or CR (100 μg/ml) for 0, 30, or 60 min. CFW treatment did not significantly alter *gfa1* transcript abundance, whereas CR exposure significantly increased *gfa1* expression at both 30 and 60 min (Fig. 3B). These findings indicate that *gfa1* transcription can respond to cell-wall perturbation, although this response is dependent on the type of cell-wall stress encountered.

To determine whether Gfa1 deficiency affects cell-wall polysaccharide composition, we quantified the major polysaccharide components of the cell wall. The Δ*gfa1* mutant contained significantly lower levels of both chitin and glucan than the WT and RT strains (Fig. 3C). The RT strain contained significantly higher glucan levels than WT (*P* < 0.05), suggesting that restoration of *gfa1* expression can alter glucan homeostasis beyond the WT level under the conditions tested. The reduction in chitin is consistent with impaired hexosamine metabolism and reduced availability of UDP-GlcNAc for chitin production. The concomitant decrease in glucan indicates that disruption of Gfa1-dependent amino-sugar metabolism is associated with broader changes in cell-wall polysaccharide homeostasis, although the mechanism underlying this effect remains to be determined. Collectively, the hypersensitivity of the Δ*gfa1* mutant to multiple cell-wall-perturbing agents, the induction of *gfa1* expression in response to CR, and the altered chitin and glucan contents demonstrate that Gfa1-dependent hexosamine metabolism is important for maintaining cell-wall integrity and polysaccharide homeostasis in *A. flavus*.

### 3.4 Gfa1 deficiency impairs sclerotial development and environmental stress tolerance

*A. flavus* forms sclerotia as specialized structures associated with environmental persistence. WT and RT strains readily produced sclerotia under the induction conditions used in this study, whereas sclerotia were barely detectable in Δ*gfa1* colonies before or after ethanol washing (Fig. 4). We next examined responses to environmental stresses on plates incubated at 37 °C. The Δ*gfa1* mutant showed marked sensitivity to sorbitol, TCEP, FeSO_4_, and itraconazole (ITR), with weaker sensitivity to tunicamycin and amphotericin B (AMB) and no obvious sensitivity to voriconazole (VOR) (Fig. 3D). These phenotypes indicate that Gfa1 deficiency compromises the ability of *A. flavus* to maintain cellular homeostasis under several environmental challenges.

**Fig. 4:**
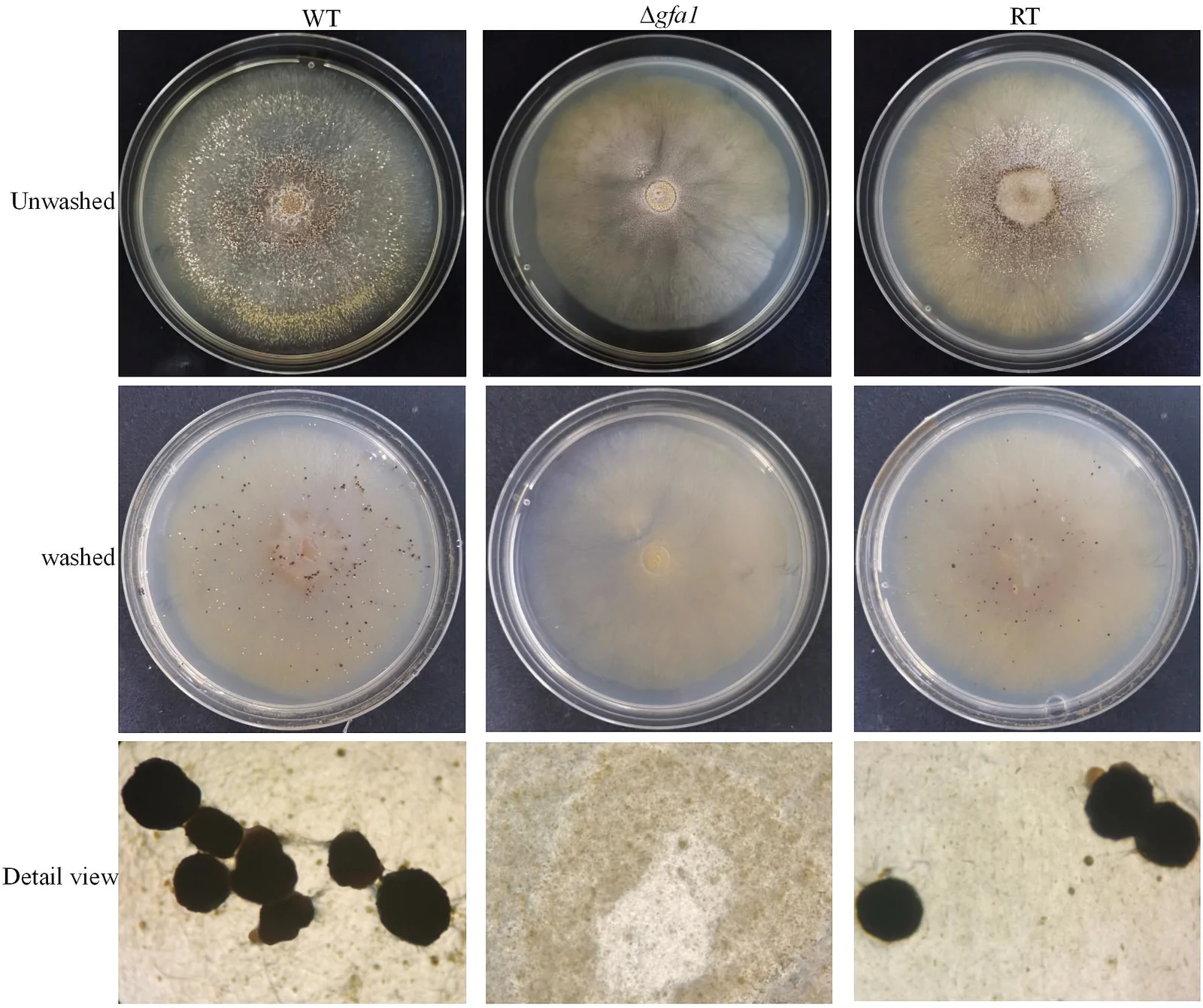
Sclerotium formation of the ∆*gfa1* mutant on minimal medium supplemented with 2.75 mM glucose and 5 mM GlcN. Conidia (10^5^) of the WT, ∆*gfa1*, and RT strains were point-inoculated onto minimal medium supplemented with 2.75 mM glucose and 5 mM GlcN and incubated in the dark at 37 °C for 10 days. Photographs were taken using a stereomicroscope.Three biological replicates were performed.

Temperature-dependent growth further revealed a pronounced phenotype at 28 °C. Relative growth inhibition at 28 °C was 54 ± 2% in Δ*gfa1* compared with 9 ± 2% and 9 ± 3% in WT and RT, respectively. At 42 °C, however, the inhibition rate of Δ*gfa1* was lower than those of WT and RT (Fig. S3A and S3B). Thus, Gfa1 deficiency caused a temperature-dependent rather than uniformly reduced thermal fitness phenotype. At 28 °C, Δ*gfa1* was also highly sensitive to osmotic, redox, and cell-wall perturbations (Fig. 3E). Because Δ*gfa1* failed to grow on CR, CFW, TCEP, and FeSO₄ at either 28 °C or 37 °C, a direct comparison of stress sensitivity between the two temperatures was not possible; however, the mutant exhibited a more pronounced growth defect at 28 °C. These results indicate that Gfa1-dependent metabolism contributes to environmental fitness under conditions relevant to the *A. flavus* lifecycle.

### 3.5 Loss of Gfa1 impairs crop-seed colonization and prevents detectable AFB1 accumulation

We next examined whether the developmental and cell-wall defects of Δ*gfa1* compromise colonization of agriculturally important substrates. Peanut and maize seeds were inoculated with WT, Δ*gfa1*, or RT strains and incubated at 28 °C for 6 days. WT- and RT-inoculated seeds showed extensive fungal growth and abundant conidiation, whereas seeds inoculated with Δ*gfa1* remained largely free of visible fungal development (Fig. 5A and B). Quantification of conidia recovered from infected seeds confirmed significantly lower fungal proliferation in the Δ*gfa1* group than in WT- and RT-infected groups (Fig. 5C and D).

**Fig. 5:**
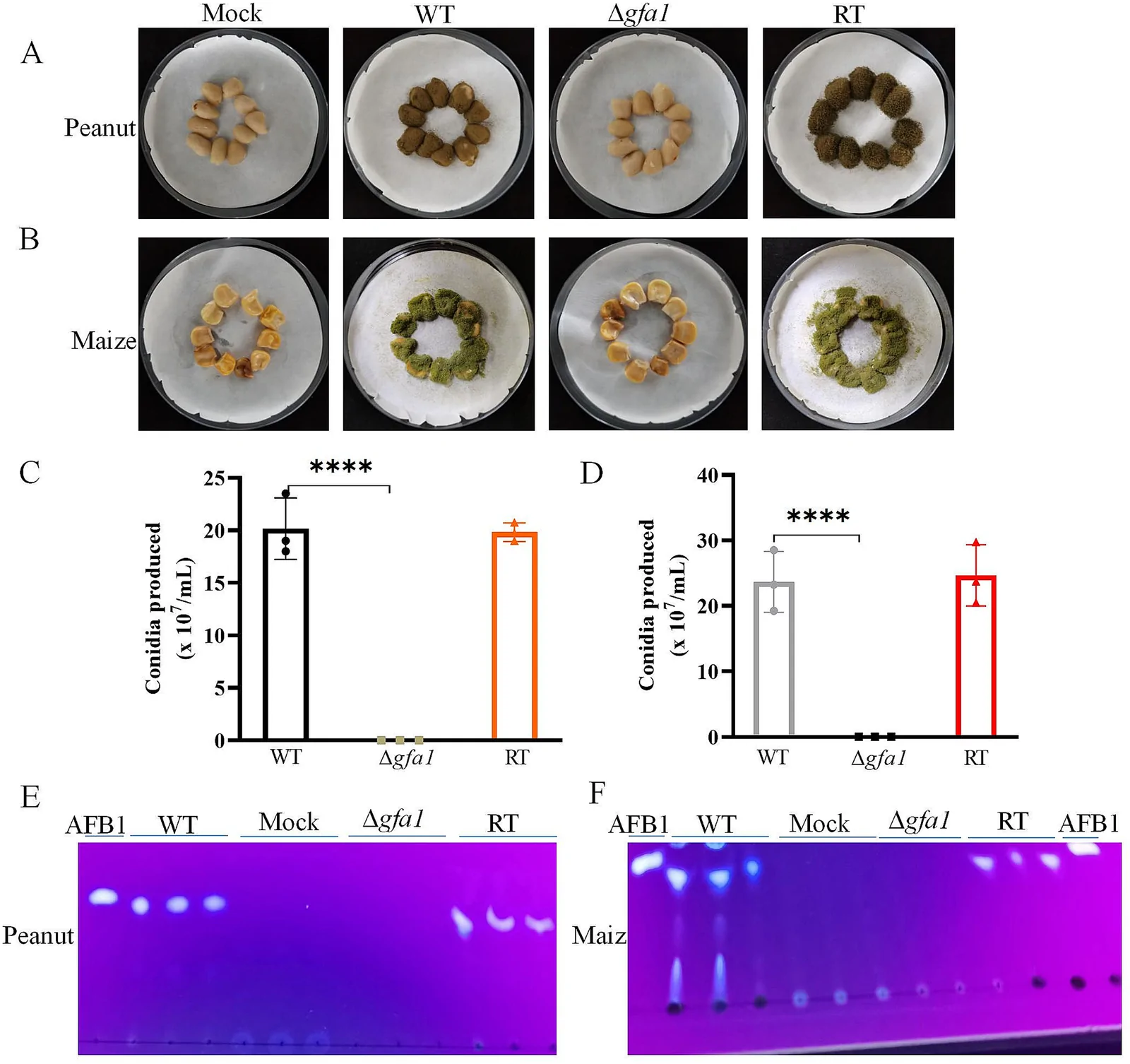
Colonization of crop seeds by the WT, ∆*gfa1*, and RT strains. (A) Conidia (10^6^) of the indicated strains were inoculated onto peanut seeds and incubated at 28 °C in the dark for 6 days. (B) Conidia (10^6^) of the indicated strains were inoculated onto corn seeds and incubated at 28 °C in the dark for 6 days. Seeds treated with 0.2% Tween-20 served as the negative control (Mock). (C) Conidia were washed from the surfaces of infected peanut seeds and quantified using a hemocytometer. (D) Conidia were washed from the surfaces of infected corn seeds and quantified using a hemocytometer. Values represent the mean ± SD from three biological replicates, each performed in triplicate. Statistical significance was determined by One-way ANOVA multiple-comparison test (**** *P* < 0.0001). (E) Thin-layer chromatography (TLC) analysis of aflatoxin B1 (AFB1) extracted from conidial suspensions washed from the surfaces of peanut seeds using equal volumes of chloroform. (F) Thin-layer chromatography (TLC) analysis of aflatoxin B1 (AFB1) extracted from conidial suspensions washed from the surfaces of corn seeds using equal volumes of chloroform.

Because *A. flavus* colonization of maize and peanut is associated with aflatoxin contamination, we next examined AFB1 accumulation. Substantial AFB1 was detected in WT- and RT-infected samples, whereas no AFB1 was detected in Δ*gfa1*-infected samples under the conditions tested (Fig. 5E and F). These findings demonstrate that Gfa1 is required for efficient colonization and proliferation of *A. flavus* on peanut and maize seeds and that Gfa1 deficiency prevents detectable AFB1 accumulation during the tested colonization process. Whether the loss of AFB1 results directly from altered aflatoxin biosynthesis or indirectly from the severe reduction in fungal growth and development remains to be determined.

### 3.6 Gfa1 deficiency strongly attenuates *A. flavus* virulence

We next examined whether Gfa1 deficiency affects host-associated fungal fitness using a *Galleria mellonella* infection model. WT, Δ*gfa1*, and RT conidia were injected into sixth-instar larvae and survival was monitored. At 72 h post-infection, survival of larvae infected with Δ*gfa1* was 96.7%, compared with 1.1% for both WT- and RT-infected groups (Fig. 6A,B). Thus, loss of Gfa1 resulted in profound attenuation of *A. flavus* virulence. Because Δ*gfa1* also displayed severe defects in growth, cell-wall integrity, development, and environmental stress tolerance, the reduced virulence is most appropriately interpreted as a consequence of impaired overall fungal fitness rather than evidence that Gfa1 functions as a dedicated virulence regulator.

**Fig. 6:**
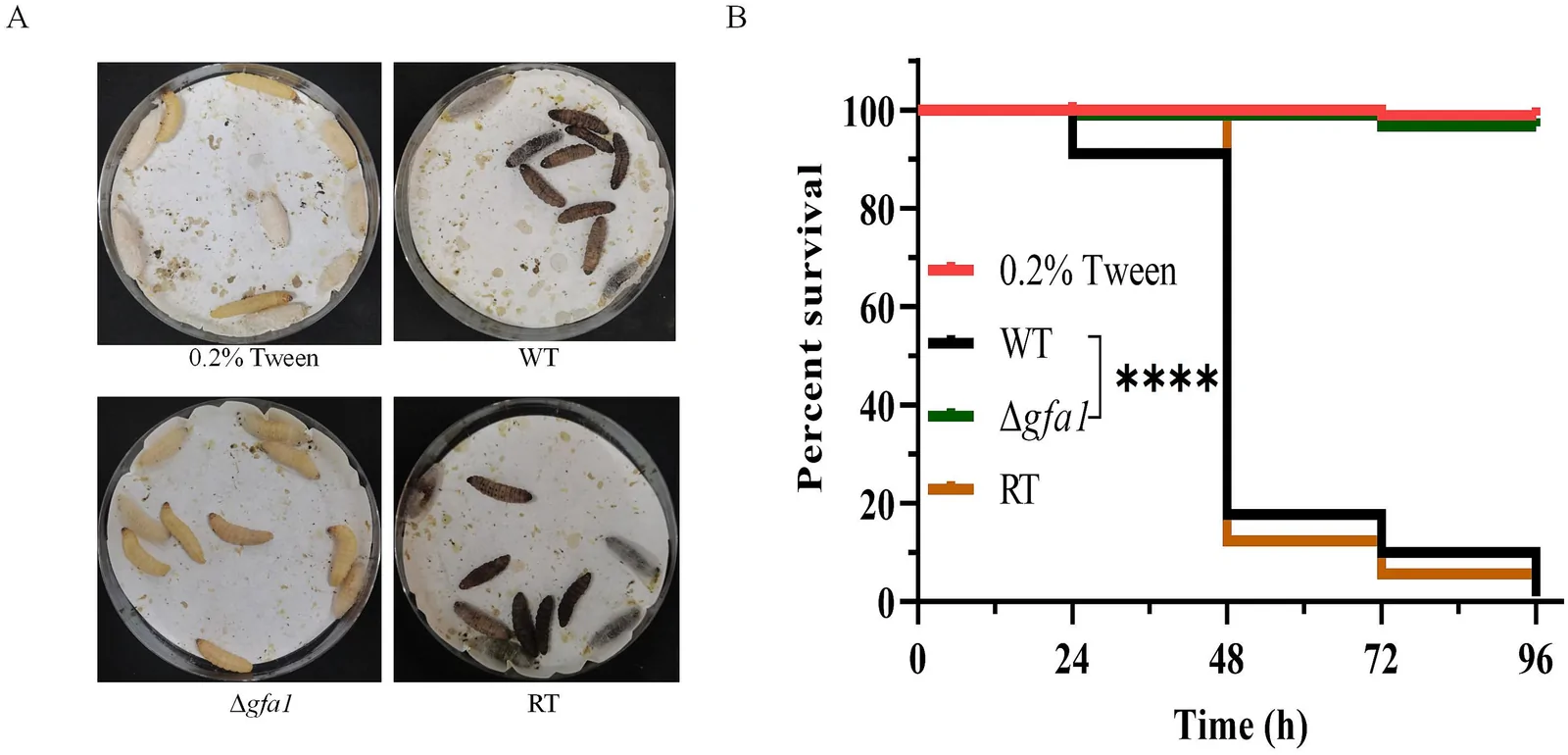
Virulence assay of the ∆*gfa1* mutant in a *Galleria mellonella* infection model. (A) Representative photographs of *G. mellonella* larvae at three days post-inoculation with conidia of the WT, ∆*gfa1*, and RT strains, or with 0.2% Tween as a control. (B) Kaplan–Meier survival curves of *G. mellonella* larvae at 24, 48, and 72 h after infection with conidia of the indicated strains. Larvae treated with 0.2% Tween served as the negative control. The incubation temperature was maintained at 37 °C throughout the experiment. Larvae were randomly divided into four groups (WT, Δ*gfa1*, RT, and control), each containing 90 larvae, and each group was further divided into three subgroups of 30 larvae (*n* = 90 per group). Statistical significance was determined by One-way ANOVA multiple-comparison test (**** *P* < 0.0001).

## 4 Discussion

The fungal cell wall is a metabolically responsive structure whose composition and organization are tightly coupled to cellular physiology and development (Gow, 2025; Gow and Lenardon, 2023; Hopke et al., 2018.). In this study, we identify Gfa1-dependent hexosamine metabolism as an important determinant of cell-wall homeostasis and developmental competence in *A. flavus*. Loss of *gfa1* caused amino-sugar auxotrophy, impaired growth and conidiation, increased sensitivity to cell-wall perturbation, and reduced cell-wall chitin and glucan. These defects extended to sclerotial development, environmental stress tolerance, crop-seed colonization, AFB1 accumulation, and host-associated fitness.

The amino-sugar auxotrophy of *A. flavus* Δ*gfa1* (Fig. S1) is consistent with the conserved role of Gfa1 in supplying the HBP intermediate required for downstream UDP-GlcNAc production. Similar phenotypes have been reported in *A. niger* and *A. fumigatus* (Ram et al., 2004; Qin et al., 2025). Importantly, our results show that amino-sugar supplementation sufficient to support mutant growth does not restore normal developmental fitness. The persistence of growth, conidiation (Fig. 2), cell-wall (Fig. 3A and C), sclerotial (Fig. 4), and stress phenotypes (Fig. 3D and E) under GlcN-supported conditions suggests that Gfa1-dependent metabolism contributes to cellular homeostasis beyond simple biomass production.

The strongest connection between Gfa1 function and cell-surface biology is reflected in the cell-wall phenotype. The Δ*gfa1* mutant was hypersensitive to CFW, CR, and caspofungin (Fig. 3A) and contained significantly less chitin and glucan than the WT and RT strains (Fig. 3C). The decrease in chitin is consistent with impaired hexosamine metabolism and reduced availability of precursors for chitin biosynthesis. The accompanying reduction in glucan suggests that perturbation of HBP activity affects broader polysaccharide homeostasis rather than a single biosynthetic branch. Similar relationships between HBP activity and cell-wall integrity have been reported in other fungi, including *A. fumigatus* and *S. cerevisiae* (Lagorce et al., 2002; Qin et al., 2025). Interestingly, the reduced chitin content of the Δ*gfa1* mutant was accompanied by increased sensitivity to CFW (Fig. 3A). This relationship contrasts with observations in *Candida albicans*, in which loss of the chitin synthase gene *chs3* results in reduced chitin content but increased resistance to CFW (Munro et al., 2007). This difference may reflect the distinct nature of the underlying defects. Whereas *chs3* deletion directly reduces chitin synthesis, Gfa1 deficiency disrupts amino-sugar metabolism upstream of chitin biosynthesis and is associated with a broader reduction in cell-wall polysaccharides, including both chitin and glucan. These findings also raise the possibility that Gfa1 contributes to the cellular response to cell-wall perturbation in *A. flavus*. In *S. cerevisiae*, cell-wall stress induces GFA1 expression and Gfa1p activity, with Gfa1p exerting substantial flux control over chitin synthesis (Lagorce et al., 2002). Similarly, cell-wall stress induces *gfaA* transcription and promotes chitin deposition in *A. niger* (Ram et al., 2004). In *A. flavus*, however, *gfa1* expression was not significantly affected by CFW but was significantly induced by CR (Fig. 3B), indicating that the transcriptional response is stressor-dependent. Thus, Gfa1 may contribute to cell-wall adaptation under specific forms of cell-wall perturbation rather than functioning as a universally stress-inducible component of the CWI response. Whether CR-induced *gfa1* expression is mediated through the CWI pathway remains to be determined.

Notably, although exogenous GlcN was sufficient to support growth of the Δ*gfa1* mutant, it did not fully restore chitin content (Fig. 3C). This finding indicates that restoration of amino-sugar availability can partially compensate for the loss of Gfa1 but is insufficient to completely normalize cell-wall polysaccharide homeostasis. A similar distinction between growth rescue and cell-wall restoration has been reported in *A. fumigatus* Δ*gfa1* (Qin et al., 2025). Thus, in *A. flavus*, the consequences of Gfa1 deficiency may extend beyond simple precursor limitation and involve broader regulation or remodeling of cell-wall composition. Collectively, these findings extend the conserved relationship between hexosamine metabolism and cell-wall homeostasis to *A. flavus*. They further suggest that Gfa1-dependent metabolism contributes to the ability of the fungus to maintain cell-wall integrity under perturbation, providing a potential mechanistic link between HBP function and the developmental and crop-associated phenotypes observed in this study.

A notable feature of the present study is the severe defect in sclerotial development. Sclerotia are specialized structures that contribute to the environmental persistence of *A. flavus*, and their formation requires substantial cellular and extracellular remodeling (Calvo and Cary, 2015; Zhao et al., 2017;Wicklow et al., 1993). The near absence of sclerotia in Δ*gfa1* (Fig. 4) therefore suggests that adequate amino-sugar metabolism and cell-wall homeostasis are required during developmental differentiation. This phenotype provides an important distinction from the recent *A. fumigatus* Gfa1 study, in which growth, germination, stress responses, and virulence were major phenotypes (Qin et al., 2025). The present findings indicate that Gfa1-dependent metabolism also contributes to an *A. flavus* developmental program with direct relevance to environmental persistence.

The temperature-dependent and stress-sensitive phenotypes further support a role for Gfa1 in environmental fitness. The mutant was particularly impaired at 28 °C (Fig. S3) and showed increased sensitivity to osmotic, redox, and cell-wall perturbation under these conditions(Fig. 3E). Because 28 °C is relevant to *A. flavus* development and secondary metabolism (Wu et al., 2023; O'Brian et al., 2007; Han et al., 2019), impaired cell-wall homeostasis may contribute to the poor performance of Δ*gfa1* under crop-associated conditions. However, the current data do not establish that Gfa1 functions as a temperature sensor or dedicated stress regulator. The contrasting phenotype at 42 °C (Fig. S3) further indicates that the effect of Gfa1 deficiency is context dependent.

The crop-seed experiments provide a direct link between Gfa1-dependent cellular homeostasis and ecological fitness. WT and RT strains extensively colonized peanut and maize, whereas Δ*gfa1* showed markedly reduced proliferation (Fig. 5A and B). No detectable AFB1 was observed in mutant-infected seeds under the conditions tested (Fig. 5E and F). These findings are relevant to the agricultural importance of *A. flavus*; however, they do not establish that Gfa1 directly regulates the aflatoxin biosynthetic pathway. The loss of detectable AFB1 could result from reduced fungal biomass, altered developmental state, or changes in the metabolic environment associated with impaired colonization. Direct measurements of aflatoxin-pathway gene expression and pathway metabolites would be required to distinguish these possibilities.

The strong attenuation of Δ*gfa1* virulence in *G. mellonella* (Fig. 6A and B) is consistent with the broad cellular and developmental defects observed *in vitro*. Similar attenuation has been reported following disruption of HBP enzymes in other fungi, including *A. fumigatus* (Malavia-Jones et al., 2026; Qin et al., 2025). Nevertheless, because Gfa1 is a central metabolic enzyme and its loss affects growth, cell-wall integrity, development, and stress adaptation, the virulence phenotype should not be interpreted as evidence for a dedicated virulence pathway. Instead, our findings support the broader concept that intact amino-sugar metabolism and cell-wall homeostasis are prerequisites for full host-associated fitness.

Taken together, our results support a model in which Gfa1-dependent hexosamine metabolism maintains the precursor supply and cellular homeostasis required for proper cell-wall organization. Perturbation of this metabolic node compromises cell-wall integrity, developmental differentiation, environmental fitness, and ultimately crop-associated colonization and pathogenicity. The study therefore extends the conserved role of Gfa1 beyond fungal viability and provides a functional connection between HBP activity, cell-surface homeostasis, sclerotial development, and the crop-associated lifecycle of *A. flavus*. Future studies should directly determine whether HBP perturbation influences aflatoxin biosynthesis independently of fungal biomass.

## CRediT authorship contribution statement

**Qijian Qin:** Writing – original draft, Visualization, Validation, Software, Methodology, Investigation, Funding acquisition, Formal analysis, Data curation. **Bin Wang:** Writing – review & editing, Software, Methodology, Formal analysis, Data curation. **Wenxia Fang:** Writing – original draft, Visualization, Validation, Supervision, Resources, Project administration, Investigation, Funding acquisition, Formal analysis, Conceptualization.

## Ethical statement

No animals were used in this study that would be covered under the regulations of the Ministry of the Interior, China.

## Declaration of competing interest

The authors declare that they have no known competing financial interests or personal relationships that could have appeared to influence the work reported in this paper.

## Data Availability

Data will be made available on request.
