# Supplementary material for: Gfa1-dependent hexosamine metabolism maintains cell-wall integrity and developmental fitness required for crop colonization in Aspergillus flavus
Source: Cell Surf. 2026 Oct 15;16:100184. doi: 10.1016/j.tcsw.2026.100184 (PMC13695157; doi:10.1016/j.tcsw.2026.100184)
Supplement: Supplementary file 1 [file mmc1.docx]

**Supplementary Materials**

Fig. S1. Growth of the ∆*gfa1* mutant on different carbon sources. Serial dilutions of conidia (10² to 10⁵) from the WT, ∆*gfa1*, and RT strains were spotted onto MM supplemented with the indicated carbon sources and incubated at 37 ℃. Plates were photographed after 48 h.

Fig. S2. Growth of the ∆*gfa1* mutant on minimal medium containing different concentrations of glucose and GlcN. Serial dilutions of conidia (10² to 10⁵) from the WT, ∆*gfa1*, and RT strains were spotted onto MM supplemented with the following combinations of carbon sources: (A) 27.5 mM glucose with 1 to 50 mM GlcN, (B) 55 mM glucose with 1 to 50 mM GlcN. Plates were incubated at 37 ℃ and photographed after 48 h.

Fig. S3. Growth of the ∆*gfa1* mutant at different temperatures. (A) Conidia (10⁵) of the WT, ∆*gfa1*, and RT strains were point-inoculated onto minimal medium supplemented with 2.75 mM glucose and 5 mM GlcN and incubated at 28°C, 37°C, or 42°C for 2 days. (B) Growth rates were calculated based on the colony diameters of the strains at 28°C or 42°C relative to those at 37°C. Data are presented as mean ± SD from three independent replicates. Statistical significance was determined by One-way ANOVA multiple-comparison test (**** P< 0.0001). Three biological replicates were performed.

**Table S 1. Primers used in this study**

| **Name** | **Sequence (5’-3’)** | **Purpose** |
| --- | --- | --- |
| P1 | aattcggatcttccagagatTGATTGAGGGGTCTAAAATTGGA | Mutant construction |
| P2 | tgaggcGGTTAAGGTATGTCAAGAGAGAGAGCG |  |
| P3 | gacataccttaaccGCCTCAAACAATGCTCTTCACC | Mutant construction |
| P4 | tagcgacaatGTCTGAGAGGAGGCACTGATGC |  |
| P5 | cctctcagacATTGTCGCTATTGCAACAGCG | Mutant confirmation |
| P6 | ttcaactgccgttcgacgatTGAGAAAGCTGCAAGTCATATACAAG |  |
| P7 | aattcggatcttccagagatTGATTGAGGGGTCTAAAATTGGA | RT construction |
| P8 | tgtttgaggcAAGAGAGGACAAAAGCGGGAA |  |
| P9 | gtcctctcttGCCTCAAACAATGCTCTTCACC | RT construction |
| P10 | ttcaactgccgttcgacgatCGGTATTGCCGATGTCGATG |  |
| P11 | TGCATAGGTCACAGTCTCGC | Mutant confirmation |
| P12 | ACCGCTAGTCTTCAACTCACC |  |
| P13 | ACACAAATAAAAATTTCGCCA | Mutant confirmation |
| P14 | GGACGCTTTGCTAATGTCTGC | Mutant confirmation |
| P15 | AGTGAGGATAGGGCGTCTAAGCA | RT-qPCR |
| P16 | TAAGTGCACCTTCAAGGGCAGT | RT-qPCR |
| P17 | ACGGTGTCGTCACAAACTGG | RT-qPCR |
| P17 | CGGTTGGACTTAGGGTTGATAG | RT-qPCR |
